# Exploration and Evaluation of Secondary Metabolites from *Trichoderma harzianum*: GC-MS Analysis, Phytochemical Profiling, Antifungal and Antioxidant Activity Assessment

**DOI:** 10.3390/molecules28135025

**Published:** 2023-06-27

**Authors:** Wassima Lakhdari, Ibtissem Benyahia, Mustapha Mounir Bouhenna, Hamdi Bendif, Hafida Khelafi, Hakim Bachir, Amel Ladjal, Hamida Hammi, Dajwahir Mouhoubi, Hanane Khelil, Taghrid S. Alomar, Najla AlMasoud, Nabil Boufafa, Fehmi Boufahja, Abderrahmene Dehliz

**Affiliations:** 1 National Institute of Agronomic Research of Algeria, Touggourt 30200, Algeria; wassimalakhdari@yahoo.fr (W.L.); midou.hamida25@yahoo.com (H.H.); dahliyacine@gmail.com (A.D.); 2 Valcore Laboratory, Biology Department, Faculty of Life and Nature Sciences, University of Boumerdes, Boumerdes 35000, Algeria; hkhelafi@hotmail.com (H.K.); ladjalamel@gmail.com (A.L.); 3 Laboratory of Biogeochemistry and Desert Environments, Department of Chemistry, Faculty of Mathematics and Material Sciences, University of Kasdi Merbah, Ouargla 30000, Algeria; benyibtissam@gmail.com; 4 Scientific and Technical Center of Research in Physical and Chemical Analysis (CRAPC), Bou-Ismail 42004, Algeria; mustapha.bouhenna@crapc.dz; 5 Department of Natural and Life Sciences, Faculty of Science, University of M’sila, M’sila 28000, Algeria; hamdi.bendif@univ-msila.dz; 6 Division of Hydraulic and Bioclimatology, National Institute of Agronomic Research (INRA), Algers 16000, Algeria; akm7.62@hotmail.fr; 7 SARL SINAL, Oran 31000, Algeria; djawahir.mouhoubi@gmail.com (D.M.); khelilhanane04@gmail.com (H.K.); nabilboufafa018@gmail.com (N.B.); 8 Department of Chemistry, College of Science, Princess Nourah bint Abdulrahman University, P.O. Box 84427, Riyadh 11671, Saudi Arabia; nsalmasoud@pnu.edu.sa; 9 Biology Department, College of Science, Imam Mohammad Ibn Saud Islamic University (IMSIU), Riyadh 11623, Saudi Arabia; faboufahja@imamu.edu.sa

**Keywords:** *Trichoderma harzianum*, bioactive metabolite, antifungal, natural products, antioxidant

## Abstract

In this study, we investigated in vitro the potential of *Trichoderma harzianum* to produce bioactive secondary metabolites that can be used as alternatives to synthetic compounds. The study focused on analyzing two extracts of *T. harzianum* using ethyl acetate and *n*-butanol solvents with different polarities. The extracts were examined using phytochemical analysis to determine the content of polyphenols, flavonoids, tannins, and alkaloids. Thin-layer chromatography (TLC) and Gas chromatography-mass spectroscopy (GC-MS) analysis were used to profile volatile organic metabolites (VOCs) present in the extracts. Furthermore, the extracts were tested for their antifungal ability using the poison food technique. For measuring antioxidant activity, the 1,1-diphenyl-2-picryl-hydrazyl (DPPH) test was used. *Trichoderma harzianum* was shown to have a significantly high content of tannins and alkaloids, with a noticeable difference between the two extracts. GC-MS analysis identified 33 potential compounds with numerous benefits that could be used in agriculture and the medicinal industry. Moreover, strong antifungal activity was identified against *Sclerotinia sclerotiorum* by 94.44%, *Alternaria* sp. by 77.04%, and *Fusarium solani* by 51.48; similarly, the IC_50_ of antioxidant activity was estimated for ethyl acetate extract by 71.47% and *n*-butanol extract by 56.01%. This leads to the conclusion that *Trichoderma harzianum* VOCs play a significant role as an antifungal and antioxidant agent when taking into account the advantageous bioactive chemicals noted in the extracts. However, to our knowledge, this is the first study in Algeria presenting detailed phytochemical analysis and GC-MS profiling of *Trichoderma harzianum* for two extracts, ethyl acetate and *n*-butanol.

## 1. Introduction

Endophytic fungi are an integral part of a plant’s mycobiome. They frequently appear in the intercellular space of their plant hosts; however, they do not appear to cause any disease symptoms [1]. Endophytes are species of microorganisms that are underutilized for the discovery of novel chemicals since they coexist asymptomatically with their hosts. They produce an array of metabolites and have the ability to produce substances that are separated from, and only produced by, higher plants [2,3]. When agrochemicals are applied poorly or excessively, phytopathogens become resistant and less susceptible [4]. Endophytes are biocontrol agents that can be utilized to control plant diseases and advance sustainable agriculture [5]. *Trichoderma* species have been the subject of extensive research and usage in biological control against phytopathogenic fungi due to their strong ability to produce significant amounts of enzymes and metabolites [6,7]. *Trichoderma* is a filamentous fungus with a wide range of uses in industry, agriculture, and the environment [8]. It has the benefit of producing a variety of bioactive metabolites and potential drug leads. *Trichoderma* is frequently used in agriculture as biofungicides and bioremediation agents because they protect the host plant throughout its entire life cycle and can therefore act as biocontrol agents [4,9,10]. Bio-efficient substances are an excellent source of potential novel therapies [11]. The demand for novel therapeutic and therapeutically beneficial chemicals is expected to continue to increase in order to face the challenges posed by rising antibiotic resistance [12]. The secondary metabolites have not been thoroughly or methodically assembled. To date, nearly 200 *Trichoderma* sp. compounds have been identified as terpenoids, polyketides, peptides, alkaloids, and lactones [7]. Furthermore, because *Trichoderma* species have a natural resistance to many agricultural agents, such as fungicides, they are integrated into pest management strategies [13].

In this field, few studies were interested in examining the potential antagonistic effects of *Trichoderma hazianum* in inhibiting the growth of the plant and pathogenic fungi [14], as the primary goal of these investigations was to protect the plant against pathogenic microorganisms, in particular the antifungal effects against *Fusarium graminarium* and *Asper-gillus terreus* [15]. Some species of this fungus have the ability to clean up contaminated environments. *Trichoderma harzianum* is one of the various methods for decreasing the detrimental effects of heavy metals on plants [16]. Sesquiterpenes and diterpenes isolated from *Trichoderma* species have been shown to exhibit anti-microbial, anti-microalgae, anti-cancer, and phytotoxic properties [17].

A wide class of carbon-based substances known as fungal volatile organic compounds (VOCs) has low molecular weights, low polarity, low boiling temperatures, and high vapor pressure [18]. These substances are frequently lipophilic and include alcohols, benzenoids, aldehydes, alkenes, acids, esters, ketones, thiols, and their derivatives, among other chemical classes [19,20].

Aside from having few adverse effects and promising therapeutic applications, bio-efficient natural compounds are a promising source of new antioxidants and antibacterial agents [11]. This study aims to investigate the bio-efficiency of the secondary metabolites of *T. harzianum* by using phytochemical analysis, TLC, and GC-MS. Furthermore, this study focuses on identifying the compounds’ capacity for antioxidant and antifungal activities as well as any potential advantages and applications.

## 2. Results

*T. harzianum* was grown and dried, and two extracts were prepared using 79.75 g of the fine powder: ethyl acetate, yielding 1.81% of the extract and *n*-butanol, yielding 1.17%.

### 2.1. Phytochemical Analysis

Phytochemical analysis was carried out on *T. harzianum* ethyl acetate and *n*-butanol extracts; a noticeable difference in the contents is shown in Table 1. A high tannin content of 1584.16 mg TAE (Tannic acid equivalent)/g DE for ethyl extract and 2192.5 mg TAE/g DE for *n*-butanol extract, followed by flavonoids of 266.18 mg QE (Quercetin equivalent)/g DE and 203.62 mg QE/g DE for ethyl extract and *n*-butanol extract, respectively. Phenolic content was estimated to be 70.54 mg GAE (Gallic acid equivalent)/g DE for ethyl acetate extract and 40.12 mg GAE/g DE for *n*-butanol extract. The alkaloid content is considered to be the lowest in our analysis, as we noted 0.83 mg NE (Nicotine equivalent)/g DE for ethyl acetate and 0.77 mg NE/g DE for *n*-butanol extract.

ANOVA analysis results show that the amount of secondary metabolites in ethyl acetate extract showed significant differences between groups F (40.028) and *p* < 0.0001. Multiple comparisons using a Tukey test indicated that there is a significant difference for the polyphenol, while the other variables (alkaloid, tannin, and flavonoid) do not show a significant difference between the subsets. The *n*-butanol showed significant differences between the groups F (340.98) and *p* < 0.0001. Multiple comparisons using Tukey’s HSD test indicate that there is also a significant difference between the groups for the variable sub-homogeneous sets. In this case, there are three subsets (1, 2, and 3) for which comparisons were made. The groups show significant variations in terms of this variable (polyphenol alkaloid, tannin, and flavonoid).

### 2.2. Thin Layer Chromatography Analysis (TLC)

Thin-layer chromatography was performed for *T. harzianum* ethyl acetate and *n*-butanol extracts. Several patterns of composition were determined based on the presence or absence of discriminant spots in the first visual inspection of the fungal extract.

**Figure 1:**
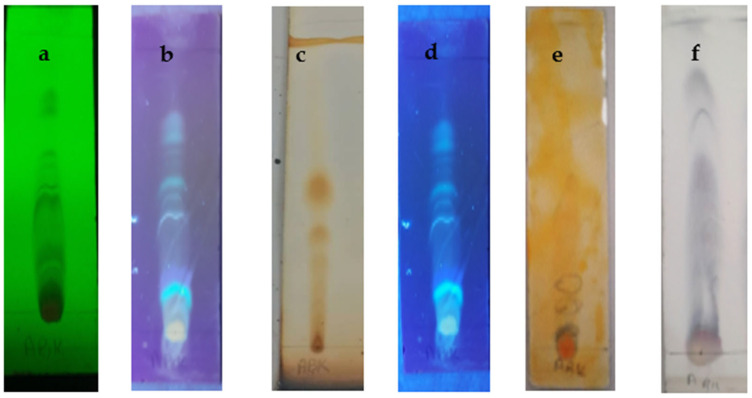
Thin layer chromatography (TLC) of *n*-butanol of *T. harzianum*. (**a**): Under UV (254); (**b**): Under UV (365); (**c**): Iodine reagent; (**d**): Aluminum AlCl_3_; (**e**): Dragendroff reagent; (**f**) Vanillin/Sulfuric acid reagent.

TLC profiles of ethyl acetate extract and the results of spraying with Vanillin/Sulfuric acid, Aluminum AlCl_3_, Iodine, and the Dragendroff test (UV 245 and 365 nm) are illustrated in Figure 1. Nine fractions were identified (F1-F9) by the Vanillin/Sulfuric test (Figure 1f), characterized by an Rf: 0.05 and 0.08 with orange color and 0.13, 0.2, 0.33, 0.36, 0.41, 0.5, and 0.7 with blue color, indicating a wide color range that shows the presence of different compounds (carbonyl compounds). Eight fractions were identified (F1–F8) by the flavonoid type aluminum test and are characterized by an Rf of 0.05 with an orange color and an Rf of 0.08, 0.13, 0.2, 0.41, 0.5, 0.62, and 0.7 with a similar blue color under UV (365) (Figure 1b). After the Dragendroff test in the system was used (Figure 1e), we noticed the presence of three fractions, including an Rf of 0.05 and 0.2 blue and an Rf of 0.13 yellow, which indicates the presence of alkaloids.

**Figure 2:**
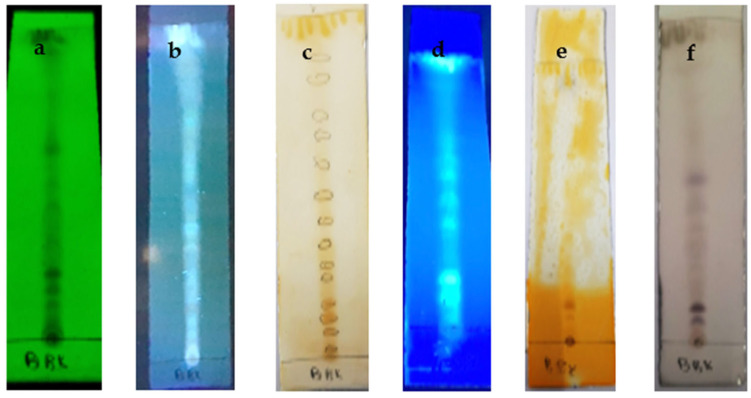
Thin layer chromatography (TLC) of *n*-butanol of T. harzianum. (**a**): Under UV (254); (**b**): Under UV (365); (**c**): Iodine reagent; (**d**): Aluminum AlCl_3_; (**e**): Dragendroff reagent; (**f**): Vanillin/Sulfuric acid reagent.

According to the results of the TLC plates of *n*-butanol extract, we noted the Vanillin/Sulfuric reagent (Figure 2f) presents 11 spots characterized by an Rf of 0.6, 0.11, 0.23, 0.4, 0.5, and 0.83 with a similar color blue. An Rf of 0.15, 0.26, or 0.57 is a yellow color. An Rf between 0.33 and 0.63 is a green color, denoting the existence of several compounds (carbonyl compounds, ketones, and phenols). The aluminum reagent presents 8 spots (Figure 2d) characterized by an Rf of 0.11, 0.4 orange color, an Rf of 0.26, and 0.34 blue color, and an Rf of 0.46, 0.5, 0.67, and 0.72 yellow color. This indicates the presence of flavonoids. The Dragendroff reagent (Figure 2e) presents three spots with an Rf of 0.09 and 0.77 in blue and an Rf of 0.069 in green.

### 2.3. GC-MS Analysis

**Figure 3:**
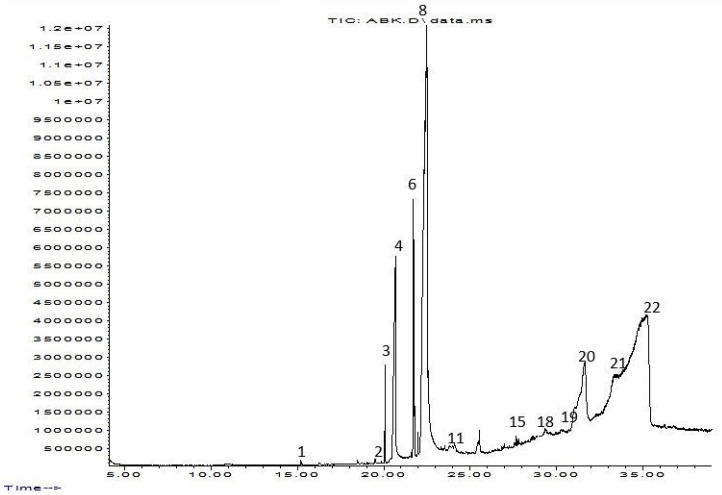
Chromatography–mass spectrometry (GC-MS) separation chromatograms for ethyl acetate extract of *T. harzianum*.

*T. harzianum* extracts by ethyl acetate and *n*-butanol were subjected to GC-MS examination. The investigations revealed an array of biomolecules (Table 2 and Table 3), and a GC-MS chromatogram of ethyl acetate extract showed 27 peaks, as shown in Figure 3. The primary components were s-Triazine trichloride (33.24%), Linoleic acid (26.95%), Ethylene sulfate (11.66%), 5-(Dimethylamino)-3,4-dihydro-4-isopropyl-4-methyl-2H-imidazol-2- one (9.90%), and Palmitinic acid (5.86%).

**Figure 4:**
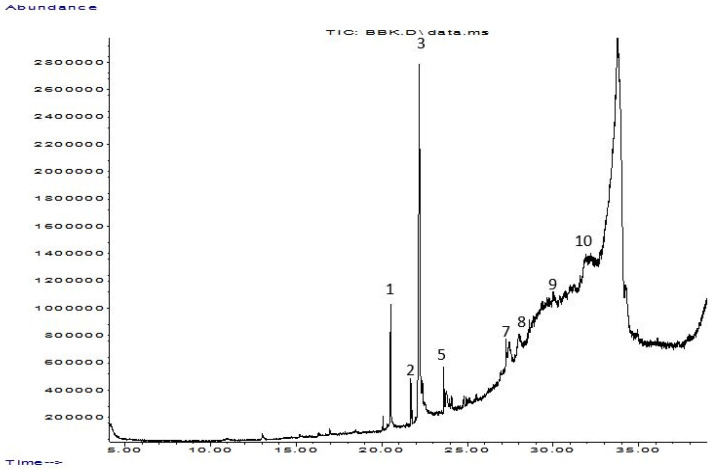
Chromatography–mass spectrometry (GC-MS) separation chromatograms for *n*-butanol extract of *T. harzianum*.

Regarding the *n*-butanol extract, it presented 10 peaks as shown in the chromatogram (Figure 4). The main constituents were Ethyl linoleate 14.05%, Glyceryl 1-oleate, diacetate 5.23%, Palmitic acid 2.72%,1H-Indazole-5-carboxylic acid, 6-nitro- 2.26%, and (Z)-11-Hexadecenal 1.54%.

### 2.4. Antioxidant Activity

DPPH radical scavenging assays were used to evaluate the antioxidant activity of *T. harzianum* ethyl acetate and *n*-butanol extracts in different concentrations. Our results (Table 4) demonstrate that the IC_50_ obtained was higher than that of ascorbic acid (0.265 mg/mL), where we noted the IC_50_ and the inhibition of ethyl acetate extract were 7.147 mg/mL, which was higher than the *n*-butanol extract with an IC_50_ of 5.415 mg/mL and 56.01%.

### 2.5. Antifungal Activity

The results of the antifungal activity of *T. harzianum* in ethyl acetate and *n*-butanol extracts are presented in Table 5. Both extracts were shown to have inhibitory activity on all tested pathogens in comparison with the control. In particular, we noted a strong activity of *n*-butanol extract against *Sclerotinia sclerotiorum* at 93.70%; however, we noted 58% against *Alternaria* sp., and ethyl acetate extract was more effective against *Alternaria* sp. with 77.04% and 51.48% against *Fusarium solani*.

Based on the results presented in Table 5, the results of the normality test of ethyl acetate against *Alternaria* sp. and *Fusarium solani* show an abnormal distribution, as the *p*-values for all tests are less than 0.05. The Dunn test for independent samples proved that the null hypothesis was rejected. We have the difference between the two groups as indicated by the results reported in Sig. ajus., and add to that the differences between an average of 100% and 25%.

For the statistical analysis of *n*-butanol extract against *Alternaria* sp., the data follow the normal distribution. Based on Tukey’s HSD test (*p* < 0.05), it can be concluded that there are significant differences in the means of the groups.

The results suggest that the data for *Sclerotinia sclerotiorum* are not normally distributed. Based on the Dunn test with *p*-values less than 0.05, the null hypothesis is rejected. We have the difference between the two groups as indicated by the results reported in Sig. ajus., and add to that the differences between an average of 100% and the control.

## 3. Discussion

*Trichoderma* species have a major impact on the production of secondary metabolites, which offer specific benefits in processes including competition, symbiosis, metal transport, growth differentiation, signaling, and mycoparasitic activity [21]. The present study investigated the potentiality of *T. harzianum* secondary metabolites and phytochemical analysis such as polyphenols, flavonoids, alkaloids, and tannins for ethyl acetate and *n*-butanol extracts of varying polarities. Da Silva et al. [22] noted polyphenols for *T. longibrachiatum* in an ethyl acetate extract of 103.62 mg g^−1^ and in an *n*-butanol extract of 140.07 mg g^−1^ in their study on flavonoids in ethyl acetate (105.07 mg g^−1^) and *n*-butanol (162.81 mg g^−1^). Our findings from this investigation on secondary metabolites were higher than those reported by [23], where he noted 3.85 ± 0.04 mg g^−1^ on polyphenol and 36.54 ± 3.17 mg g^−1^ on flavonoids, whereas the total phenolic compounds and total flavonoids content of *T. harzianum* were estimated to be 12.18 and 6.33 mg QE/100 mL, respectively. According to [24], *Trichoderma* metabolites have displayed beneficial effects on plants, increasing plant growth and development and inducing defense responses to abiotic stresses and pathogens [25]. The presence of flavonoids and phenolic compounds in the extract is associated with the growth of *Trichoderma* sp. [26]. In response to *Trichoderma* species, phenolic compound accumulation has been linked to biochemical defense against plant diseases. Furthermore, the increased synthesis of phenols and flavonoids has a direct effect on antioxidant activity by acting as free radical scavengers and contributing to cell wall formation, which defends plants from instances of biotic stress [27,28,29]. Flavonoids serve as endogenous regulators of auxin movements and mediate developmental regulation [30,31]. Moreover, polyphenolic compounds have been recognized to possess pharmacological properties such as antioxidative, hepatoprotective, antibacterial, anti-inflammatory, anticancer, and potential antiviral properties [32]. Polyphenolic substances known as tannins and alkaloids have astringent, diuretic, anti-inflammatory, antiseptic, antioxidant, and hemostatic characteristics. The treatment of gastric and duodenal cancers is another application for them [33,34]. The presence of alkaloids and tannins was confirmed by [35] for *Trichoderma* sp.; however, [36] noted the presence of tannins and the lack of alkaloids for *T. harzianum*. Ref. [37] reported that *T. harzianum* and *T. viren* both contain only alkaloid compounds. Ecologically, the accumulation of alkaloids is an important chemical defensive strategy used by plants to adapt to environmental stresses such as endophytes, pathogens, and herbivores [38,39].

Thin-layer chromatography was performed for ethyl acetate and *n*-butanol extracts of *T. harzianum*. The sequential extraction of the extract obtained revealed 1.81% and 1.17% extraction percentages, respectively. The presence of several bioactive biomolecules was detected using thin-layer chromatography in *T. harzianum*. Spraying with different reagents showed a wide range of colors that denote the presence of various compounds (carbonylated compositions). The TLC profile suggests interesting chemical compositions that have bioactive compounds related to abiotic activities present in the ethyl acetate and *n*-butanol extracts.

In the present study, *T. harzianum* extracts were subjected to Chromatography-mass spectrometry (GC-MS), showing a profile of secondary metabolites that covers all the numerous substances that a fungus may make on a certain substrate, including antibiotics and other outwardly directed substances [40]. 22 compounds were identified in ethyl acetate extracts and 11 in *n*-butanol extracts. The major compounds are fatty acid, ester, aldehyde, hydrazide, pyrazine, imidazole, triazine, and fatty amide. A few of the compounds found were similar between the two extracts.

Researchers have found that a large number of fatty acids have antimicrobial activities and the potential for medicine in nutritional therapy [41]. Some fatty acids have the potential for antituberculosis therapy [42]. Linolenic acid, linoleic acid, and oleic acid possess substantial activity against *R. solani*. In recent work [13], the antibacterial activity of linolenic acid was demonstrated, while [43] showed that linoleic acid and oleic acid possessed insecticidal activity against the fourth-instar *Aedes aegypti* larvae. Recently, linoleic acid and glycerol monolinolate have been reported to have sporogenic activities, enhancing the asexual spores of *Alternaria* tomato [44] and *Sclerotinia fructicola* [45], respectively. Thus, linoleic acid and its congeners may have important regulatory roles in sexual as well as asexual reproductive processes in fungi [46]. Microorganism extracts are considered another alternative to traditional fungicides and pesticides as they produce bioagents that are effective against bacteria and fungi. Ref. [1] mentioned that *T. harzianum* can be used as a biopesticide against different insect pests, and [47] reported that the compounds Hexadecenoic acid and 7,10-Octadecadienoic acid can be characterized as pesticides, nematicides, and insecticides. The efficacy of pentadecanal and pentadecanoic acids as anti-biofilm agents has been recently reported against different bacterial strains, and [48] reported that Glyceryl 1-oleate and diacetate have antifungal and antimicrobial activity.

An ethyl acetate extract of *T. harzianum* showed the presence of the compound massoia lactone, characterized as a new type of biosurfactant that can be produced by *Aureobasidiumpullulans* [49], *Cyberlindnera samutprakarnensis* [50], and *Candida* sp. [51]. Some biosurfactants are also biologically active compounds with antifungal, antitumor, and anticancer proliferative activities [52]. Massoia lactone has strong anti-fungal activity and many modes of action and may be a good option for development as an efficient and environmentally friendly bio-fungicide [53].

Pyrazines are aromatic heterocyclic nitrogen-containing compounds that are important flavoring agents in many raw and roasted food products. Most alkyl pyrazines found in food result from the condensation of aldehydes. Pyrazines are also found to be produced by a wide variety of insects and play a role as pheromones [54]. For instance, some fungi imitate flowers to draw in insects that act as vectors for the spread of fungi [55]. Due to the aromatic properties of pyrazines, they have many uses in the flavor and fragrance industries [56], and 2-methoxy-3-butylpyrazine can be used as an ingredient in various perfumes [57].

Searching for strains capable of producing active ester hydrolases such as ethylene glycol against PET films is an important step in worldwide recycling [58]. Producers such as *Trichoderma* [59], *Aspergillus* [60], *Penicillium* [61], *Alternaria* [62], and *Fusarium* [52] are widely used in biotechnological applications and organic chemistry. Their use as a model for plastic biodegradation and chemical analyses has been reported in many studies [63,64].

Modern heterocyclic chemistry relies on the synthesis, reactions, and biological characteristics of substituted imidazoles. Compounds possessing an imidazole ring system have been found to exhibit a number of pharmacological properties, such as anticancer [65], carboxypeptidase [66], anti-aging [67], anti-fungal [68], anti-bacterial [69], anti-diabetic [70], and anti-malarial [71].

Given its broad spectrum of biological applications, s-triazine has attracted a considerable amount of attention from chemists due to its rich source of pharmacological activities, including antibacterial [72,73,74], antimalarial [73], antiprotozoal [75], antifungal [76], anticancer [77], antimycobacterial [78], and antiviral [79].

Regarding the activity shown by the compounds, it is known that secondary metabolites possessing aldehyde groups, especially unsaturated aldehydes, are bioactive and were found to be inhibitory to seed germination [80,81], pollen germination [82], pathogenic fungi [83], and bacteria [84].

In addition, hydrazide derivatives are available in numerous bioactive atoms and show a wide variety of biological activities. Hydrazide has been demonstrated to possess antibacterial [85], anticancer [86,87], antitubercular [88,89], anti-inflammatory [90], and antifungal activity [91].

Strategies can be developed to use these fungi for the exploitation of bioactive compounds. In addition, the use of endophytes as potential factories for the production of secondary metabolites might revolutionize agricultural, pharmaceutical, and biotechnological research in the near future [92].

An *n*-butanol extract of *T. harzianum* showed the presence of (Z)-11-Hexadecenal. Ref. [93] mentioned that (Z)-11-hexadecenal, a major component of *M. separata* sex pheromone, was found to attract early-instar larvae of *M. separata*, and this could aid in the development of olfaction-based methods for controlling *M. separata* crop damage in the larval stage. The use of (Z)-11-Hexadecenal pheromone is expected to reduce the use of chemical pesticides in *C. perspectalis* [94]. Numerous studies have mentioned biperiden as an anticholinergic drug [95].

Erucylamide is a known compound with recognized antimicrobial activity [96]. The antimicrobial effect of (Z)-docos-13-enamide, one of the most abundant constituents detected in the studied fractions known as erucamide, has been studied by [97], who detected the formation of hydrogen bridges between erucamide and amino acid residues of tubulin and glucosamine-6-P synthase, which could explain their anthelmintic and antibacterial actions.

The compounds without traceable or known biological activity may be novel ones that should be further investigated to reveal their functions. The varying biological activities of the bioactive compounds may account for the treatment of health disorders such as high blood pressure, diabetes, asthma, fever, and cancer [98].

In the present study, the ethyl acetate and *n*-butanol extracts of *T. harzianum* were evaluated for antifungal activity against three phytopathogenic fungi: *Sclerotinia sclerotiorum*, *Alternaria* sp., and *Fusarium solani*. Similar to our results, *Trichoerma* species have been used with efficacy to treat plant diseases brought on by *Fusarium* [99], *Alternaria,* and *Sclerotium* [100]. The potency of metabolites derived from *Trichoderma* species as antifungal agents against plant diseases was reported by Živković et al. [101]. For example, they inhibited *Fusarium solani* (74.4%), *Alternaria solani* (70.0%), *Pythium aphanidermatum* (67.7%), and *Macrophomina phaseolina* (50.0%). The bioactivity of the sample secondary metabolites from *T. harzianum* shows that all four *Trichoderma* species significantly inhibited the mycelial growth of the four pathogens, *Sclerotinia sclerotiorum* [102]. Strains of *Trichoderma* such as *T. harzianum*, *T. hamatum*, *T. asperellum*, and *T. atroviride* are applied for the control of phytopathogens and also as plant growth promoters in agriculture and inhibit mycelia growth as well [103]. The metabolite of *T. harzianum* produced in agar culture inhibited the growth of all 3 pathogenic fungi tested in vitro (Table 1). *Trichoderma* species are known to produce a number of antibiotics, such as trichodermin, trichodermol, harzianum A, and harzianolide [104]. Our study demonstrated the involvement of volatile metabolites in the inhibition of pathogenic fungi. The secondary metabolites produced by the fungal strains have broken down into various classes of antifungal compounds and contribute to antifungal activity, as shown in the GC-MS analysis of the two extracts. Our results implicated that the content of phenols and flavonoids, as well as the productivity of these aforementioned compounds by *T. harzianum,* could be responsible for the anti-fungal activity, modulators of pathogenicity, and activators of plant defense [26].

The antioxidant activity of ethyl acetate and *n*-butanol in *T. harzianum* was studied. Correspondingly, the antioxidant activity of *T. longibrachiatum* in the ethyl acetate extract was estimated to be 3.77 mg g^−1^ and in the butanoic extract, 304.18 mg g^−1^ [22]. However, [105] noted an antioxidant activity of 54.61% for the ethyl acetate extract of *Trichoderma* sp., whereas [51] noted 72.72% for *T. longibrachiatum* and 53.43% for *T. subviride*. The extracts obtained from *T. harzianum* exhibit strong antioxidant activity [106]. The antioxidant activity of the stable radical DPPH demonstrates the ability of molecules from the fungal extracts to scavenge these radicals. The high activity can be linked to the presence of numerous secondary metabolites, as shown in the phytochemical and GC-MS analyses [107,108,109]. Secondary metabolite analysis provides information for developing new pharmacological agents that can act as antioxidants. These active compounds can be used as sources of natural antioxidants and replace extraction from actual plants. The majority of antioxidants used today are produced industrially, although they are responsible for liver damage and carcinogenesis [110]. Contrarily, antioxidants of natural origin, such as those created by endophytes, have not been proven dangerous, particularly because of the rich diversity of life and the evolution of biochemistry [111].

## 4. Material and Methods

### 4.1. Chemical and Fungi Material

All chemical material and endophytic fungi were provided by the Biopesticides Laboratory, INRAA, Touggourt. The chemicals used are *n*-butanol, ethyl acetate, Folin-Ciocalteu reagent, sodium carbonate, sodium nitrite, aluminum chloride, hydrochloric acid, vanillin, phosphate buffer, BCG solution, chloroform, Hexane, acetic acid, Dragendroff, dimethyl sulfoxide (DMSO), 1, 1-diphenyl-2-picryl-hydrazyl (DPPH), and methanol.

The endophytic fungi evaluated in this study are *T. harzianum*, which was isolated from soil collected from the southeast of Algeria in Sidi Mehdi (33°4′18.27″ N 6°5′43.14″ E). Three pathogenic fungi, *Sclerotinia sclerotiorum* and *Alternaria* sp., were isolated from *Solanum tuberosum* l and collected from Ouadsouf (33°22′4.12″ N 6°51′5.91″ E), and *Fusarium solani* was isolated from *Solanum lycopersicum* collected from Touggourt (33°06′00″ N 6° 04′00″ E).

### 4.2. Extaction Method

The extraction of secondary metabolites from *T. harzianum* was done using the modified method of [112]. After the cultivation of fungus under fermentation conditions in a liquid medium of PDB for 14 days, the mycelia were separated from the broth through vacuum filtration. After drying, the mycelia were placed in Soxhlet, *n*-butanol, and ethyl acetate extraction was performed for 2 h, then the extract was evaporated by a rotary evaporator and stored until use.

### 4.3. Phytochemical Screening

Phytochemical analyses of polyphenols, flavonoids, alkaloids, and tannins were performed for ethyl acetate and *n*-butanol extracts of *T. harzianum*. All tests were done in triplicate [113]. All analyses were performed in the Biopesticides Laboratory, INRAA, Touggourt.

#### 4.3.1. Quantification of Polyphenol Content

The Folin—Ciocalteu reagent was used to detect total polyphenols spectrophotometrically using the colorimetric technique [114]. This evaluation is based on a measurement of the total number of hydroxyl groups present in the extract. In a nutshell, 200 µL of each extract, 800 µL of a 7.5% sodium carbonate solution, and a combination of 1 mL of reactive Folin-Ciocalteu that had been diluted 10 times were added to glass hemolyze tubes. For 30 min, the tubes were swirled and held. The absorption was focused at 765 nm. In parallel, a series of etaloning tests using various concentrations of acetic acid (0 to 1000 g/mL) were carried out under the same operating conditions [115].

#### 4.3.2. Quantification of Flavonoids Content

A method for measuring flavonoids was used based on the aluminum chloride and oxygen atmospheres found on carbons 4 and 5 of the flavonoids coming together to form a very stable combination [116]. The procedure utilized, with a few modifications, is based on those explained by Zhishen et al. [117] and Kim et al. [118]. In a glass hemolysis tube, 120 μL of NaNO_2_ at 5% was mixed with 400 μL of extract, etalon, or distillate water for reference. 120 µL of 10% AlCl_3_ was added after 5 min, and the mixture was thoroughly mixed. After 6 min, 800 μL of NaOH at 1 M was added to the solution. The absorbance was measured immediately at 510 nm against the reference. A quercetin methyl ester solution was prepared. The etaloning curve can be tracked using various solutions derived from the mother solution that range in concentration from 0 to 1000 g/mL.

#### 4.3.3. Quantification of Tannis Content

We combined the HCl approach with the vanillin method. Tanins’ capacity to transform into red anthocyanidols by interaction with vanillin explains how this approach depends on the reactivity of vanillin with the terminal grouping of the TCs’ flavonoids and the generation of red complexes [119,120]. The vanillin technique published by [121] was used to determine the concentration of condensed tannins. 1500 μL of the vanillin/methanol solution at 1% were added with 50 μL of each extract, and the mixture was stirred well. Hydrochloric acid (HCl) at a concentration of 4% was then added in a volume of 750 μL. The completed combination was allowed to rest for 20 min at room temperature. The absorbance was measured at 550 nm. Different concentrations ranging from 0 to 1000 g/mL prepared from the catalytic mother solution will enable the tracing of the etaloning curve.

#### 4.3.4. Quantification of Alkaloids Content

Accurately measured aliquots (0.4, 0.6, 0.8, 1, and 1.2 mL) of fungi extract were transferred to different separatory funnels. Then 5 mL of pH 4.7 phosphate buffer and 5 mL of bromocresol green were taken, and the mixture was shaken with extracts of 1, 2, 3, and 4 mL of chloroform. The extracts were then collected in a 10 mL volumetric flask and then diluted to adjust the solution with chloroform. The absorbance of the complex in chloroform was measured at 470 nm in a UV-spectrophotometer (SHIMADZU UV-1800, Kyoto, Japan) against the blank prepared as above but without a standard [122].

### 4.4. Thin Layer Chromatography

Apart from gas chromatography, thin-layer chromatography (TLC) is the most efficient technique for separating and identifying fungus composition. When a fraction is investigated by TLC, it is possible that a fraction that seemed homogenous by gas chromatography really included many components [123].

Both ethyl acetate and *n*-butanol extracts were examined by TLC using the solvent systems: for the ethyl acetate extract, Chloroform/Hexane/acetic acid (8:2:0.1) and for the *n*-butanol extract, *n*-butanol/water/acetic acid (4:2:0.1) on silica gel G plates (20 × 10 cm). One set of duplicated TLC plates served as the reference chromatogram. Spots and bands were visualized by UV irradiation (254 and 365 nm) and by spraying with the following reagents: Vanillin/Sulfuric acid, Aluminum AlCl_3_, and Dragendroff [124]. They are characterized by a retention factor (Rf).

### 4.5. Gas Chromatography-Mass Spectroscopy GC-MS

Separation of hydrocarbons and other volatile compounds from ethyl acetate and *n*-butanol extracts of *T. harzianum* was determined with a GC (C.R.A.P.C., Bou-Ismail, Algeria). Chromatograph: Hewlett-Packard Agilent 6890 plus Mass spectrometer: Hewlett Packard Agilent, CA, USA. GC–MS analyses were done with an ionization energy of 70 eV.

The putative identification of volatile metabolites was performed by three different chromatographic runs using three different capillary columns with different stationary phases. The putative identification of volatile metabolites was performed in capillary columns with different stationary phases. With a non-polar column (HP-5MS) of 30 m, 0.25 mm, and 0.25 µm, the oven program had an initial temperature of 60 °C for 5 min, 10 °C/min up to 300 °C, and isotherm for 10 min; injector temperature was kept at 250 °C (splitless), and detector temperature was at 280 °C.

### 4.6. Antifungal Activity

The Poison Food Technique method was used to assess the antifungal activity. The activity of the two extracts was evaluated against three pathogens: *Sclerotinia sclerotiorum*, *Alternaria* sp., and *Fusarium solani*. Each extract was reconstituted (4 mg/mL) in dimethyl sulfoxide (DMSO) in 20 mL PDA, and a 6 mm pathogen disc was put in the center of each Petri plate. The plates were then incubated at 27 °C until the control plate reached the edges. The diameters of the inhibition zones were measured in centimeters. The activity was performed in triplicate, and all treated plates were compared with the controls to calculate the inhibition percentage of growth of *T. harzianum* [125].

The minimal inhibitory concentration (MIC) was defined as the lowest concentration, determined by performing a series of dilutions of 100%, 50%, and 25%. The lowest concentrations showing a clear zone of inhibition were taken as the MIC [126].

Mycelia growth was monitored by measuring colony diameter in centimeters. The inhibition percentage of mycelia growth is calculated by the following formula [127]:

Inhibition percentage I% = (C_1_ − C_2_)/C_1_ × 100

C_1_: Diametrical growth of the control.

C_2_: Diametrical growth of the fungus in the presence of a precise concentration (C) of the extract.

### 4.7. Antioxidant Activity

The free radical scavenging activities were assessed for ethyl acetate and *n*-butanol extracts of *T. harzianum*, and the extracts were measured using 1,1-diphenyl-2-picryl-hydrazyl (DPPH). Serial dilutions were made to check the IC_50_. An extract concentration of 0.1 mg/mL^−1^ of endophytic crude extract dissolved in methanol (75 μL) was mixed with 250 μL of a methanolic solution containing 1,1-diphenyl-2-picrylhydrazyl (DPPH, Sigma, St. Louis, MO, USA). radicals obtained from a fresh DPPH solution that was prepared by mixing 24 mg of DPPH in 100 mL of methanol and storing it at 20 °C before use. After aggressively shaking the mixture and letting it stand in the dark for 30 min, the absorbance was measured at 517 nm. Ascorbic acid was used as the standard antioxidant. All readings were taken in triplicate [128]. The following formula was used to determine the capacity to scavenge the DPPH radical equation:

DPPH scavenging (%) = (A_0_ − A_1_)/A_0_ × 100

A_0_: Absorance of the control reaction.

A_1_: Absorance in the presence of the sample.

### 4.8. Statistical Analysis

In a statistical descriptive study of the inhibition rate of fungi, the key characteristics of a dataset that includes measurements of the inhibition rate of fungi are summarized and described [22]. The software IBM SPSS Statistics (Statistical Package for the Social Sciences) v.24 for Windows was used to perform parametric (ANOVA followed by Tukey’s HSD test) and non-parametric (Kruskal–Wallis followed by Dunn test) tests [129,130,131]. All the series were first checked for normality (Kolmogorov–Smirnov test) and equality of variance (Bartlett test) to decide which tests were more appropriate.

## 5. Conclusions

*T. harzianum* biosynthesizes biopotent products and has varied nutritional, industrial, and medical applications. A gas chromatography-mass spectroscopy examination demonstrates the presence of 33 compounds in total. The present study shows a variety of bioactive compounds in *T. harzianum* extracts that provide beneficial effects. Furthermore, it possesses the distinctive ability to produce bioactive secondary metabolites for human use as proficient therapeutic agents against various diseases. Ethyl acetate and *n*-butanol extracts demonstrated different levels of polyphenols, flavonoids, and alkaloids. The antifungal inhibitory effect of ethyl acetate and *n*-butanol extracts obtained in this study against pathogenic fungi might be a result of antifungal antibiotics present in secondary metabolites. *T. harzianum* possesses the advantage of large-scale production of diverse bioactive metabolites and potential drug leads, which is not always possible in plants. They are widely used in agriculture as biofungicides and bioremediation agents.

## Figures and Tables

**Table 1:** Phytochemical analysis of *T. harzianum* extracts.

	Ethyl Acetate Extract	*n*-butanol Extract	Curve Equation	R^2^
Total phenolics (mg GAE/g DE)	70.54 ± 5.92	40.12 ± 1.21	ABS = 0.009x + 0.194	0.999
Total flavonoids (mg QE/g DE)	266.18 ± 15.11	203.62 ± 4.28	ABS = 0.001x + 0.031	0.996
Total alkaloids (mg NE/g DE)	0.83 ± 0.11	0.77 ± 0.10	ABS = 0.0441x + 0.1002	0.999
Total tannins (mg TAE/g DE)	1584.16 ± 407.22	2192.5 ± 50	ABS = 4 × 10^−5^x + 0.039	0.995

GAE: Gallic acide equivalent. QE: Quercetin equivalent. NE: Nicotine equivalent. TAE: Tannic acid equivalent. DE: Dry extract.

**Table 2:** Chemical composition of ethyl acetate extract of *T. harzianum* by GC-MS.

N°	RT	Compound	Structure	Molecular Formula	MW g/mol	Peak Area %	Compound Nature
1	15.201	Massoialactone	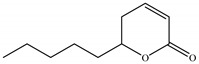	C_10_H_16_O_2_	168.23	0.05	Pyranone
2	19.511	Pentadecanoic acid	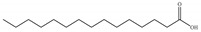	C_15_H_30_O_2_	242.39	0.06	Fatty Acid
3	20.071	Palmitic acid, methyl ester	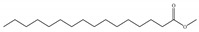	C_17_H_34_O_2_	270.45	0.59	Fatty Acid
4	20.654	Palmitinic acid	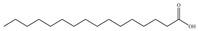	C_16_H_32_O_2_	256.4241	5.86	Fatty Acid
5	21.282	Capric acid	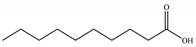	C_10_H_20_O_2_	172.26	0.14	Fatty Acid
6	21.717	Methyl linolelaidate	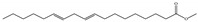	C_19_H_34_O_2_	294.48	3.23	Fatty Acid
7	21.974	Methyl stearate	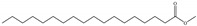	C_19_H_38_ O_2_	298.503	0.33	Fatty Acid
8	22.477	Linoleic acid	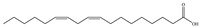	C_18_H_32_ O_2_	280.45	26.95	Fatty Acid
9	23.523	9,17-Octadecadienal, (Z)	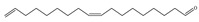	C_18_H_32_O	264.44	0.73	Aldehyde
10	23.523	14-Methyl-8-hexadecyn-1-ol	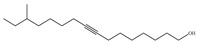	C_17_H_32_O	252.44	0.46	Fatty Alcohol
11	24.089	Ethyl linoleate	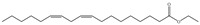	C_20_H_36_O	308.49	0.71	Fatty Acid
12	26.975	(16S)-12,16-epoxy-6.beta.-hydroxy-17(15-16)-abeo-abieta-8,12-diene- 3,11,14-trione	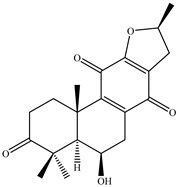	C_20_H_24_O_5_	344.41	0.20	Ketone
13	27.558	Ethyl 2-(2-chloroacetamido)-3,3,3-trifluoro-2-(2-fluoroanilino) propionate	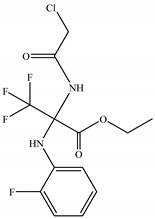	C_13_H_13_ClF_4_N_2_O_3_	356.70	1.58	Ester
14	27.667	Allyl 2-Nitrophenylpyruvate Oxime	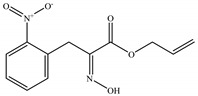	C_12_H_12_N_2_O_5_	264.24	0.16	Ester
15	27.804	3-Méthyl mercaptopropanal	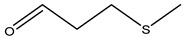	C_4_H_8_OS	104.17	0.23	Aldehyde
16	28.610	N(2)-[bis(hexafluoromethyl)methylene] oxamoyl hydrazide	/	/	/	1.03	Hydrazide
17	28.833	1-Monolinoleoylglycerol trimethylsilyl ether	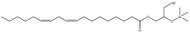	C_24_H_46_O_4_Si	426.71	0.80	Ester
18	29.330	2-Methoxy-3-methylpyrazine	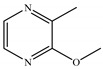	C_6_H_8_N_2_O	124.14	0.98	Pyrazine
19	30.273	1-Monolinoleoylglycerol trimethylsilyl ether	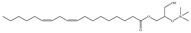	C_24_H_46_O_4_Si	426.71	1.12	Ester
20	31.627	5-(Dimethylamino)-3,4-dihydro-4-isopropyl-4-methyl-2H-imidazol-2- one	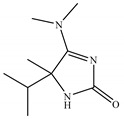	C_9_H_17_N_3_O	183.26	9.90	Imidazole
21	33.348	Ethylene sulfate	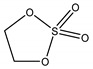	C_2_H_4_O_4_S	124.11	11.66	/
22	35.200	s-Triazine trichloride	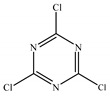	C_3_Cl_3_N_3_	184.40	33.24	Triazine

**Table 3:** Chemical composition of *n*-butanol extract of *T. harzianum* by GC-MS.

N°	RT	Compound	Structure	Molecular Formula	MW g/mol	Peak Area %	Compound Nature
1	20.52	Palmitic acid	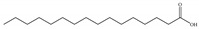	C_16_H_32_O_2_	256.42	2.72	Fatty Acid
2	21.70	Ethyl linoleate	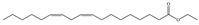	C_20_H_36_O_2_	308.51	0.44	Fatty Acid
3	22.19	Linoleic acid	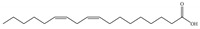	C_20_H_36_O_2_	308.50	14.05	Fatty Acid
4	1	(Z)-11-Hexadecenal	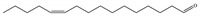	C_16_H_30_O	238.40	1.54	Aldehyde
5	23.62	Biperiden	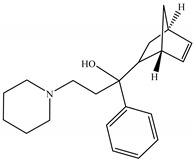	C_21_H_29_NO	311.46	0.54	Alcohol
6	27.25	Erucylamide	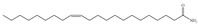	C_22_H_43_NO	337.58	1.43	Fatty amide
7	27.45	6-nitro-1H-indazole-4-carboxylic acid	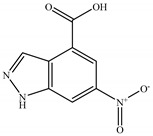	C_8_H_5_N_3_O_4_	207.15	2.26	Fatty Acid
8	28.007	Monolinolein TMS	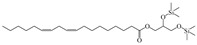	C_27_H_54_O_4_Si_2_	498.88	1.47	Ester
9	29.33	Ethyl 2-(2-chloroacetamido)-3,3,3-trifluoro-2-(3-fluoroanilino)propionate	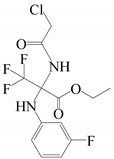	C_13_H_13_ClF_4_N_2_O_3_	356.70	1.13	Ester
10	31.91	Glyceryl 1-oleate, diacetate	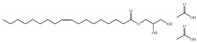	C_25_H_48_O_8_	476.65	5.23	Fatty Acid

**Table 4:** Inhibition percentage IC50 of ethyl acetate and *n*-butanol mL extracts of *T. harzianum*.

DPPH Assays	IC_50_ mg/mL
Ethyl acetate extract	7.147 ± 0.181
*n*-butanolextarct	5.415 ± 0.238
Ascorbic acid	0.265 ± 0.007

±: standard deviation.

**Table 5:** Inhibitory activity of *T. harzianum* extracts against pathogenic fungi.

Extracts	Concentration	Pathogenic Fungi %		
		*Alternaria* sp.	*Fusarium solani*	*Sclerotinia sclerotiorum*
Ethyl acetate extract	100%	77.04 ± 0.83 (a)	51.48 ± 1.09 (b)	ND
	50%	71.48 ± 3.12 (a)	9.63 ± 0.39 (c)	ND
	25%	44.07 ± 1.7 (b)	1.85 ± 0.17 (c)	ND
*n*-butanol extract	100%	58 ± 10.13 (a)	ND	93.70 ± 0.02 (a)
	50%	64 ± 5.05 (b)	ND	94.44 ± 0.01 (a)
	25%	68 ± 12.72 (b)	ND	61.48 ± 2.38 (b)
Fungicide (Fosetyl-Alumium 310 g/L)	100%	55 ± 0.80	35 ± 0.9	60 ± 1.41
	50%	15 ± 0.25	31 ± 0.88	42 ± 1.55
	25%	0	0	0

ND; not determined. The letters a, b, and c indicate the significant differences between the groups, according to Tukey’s multiple comparison test with a level of significance of 0.05. Means with the same letter are not significantly different.

## Data Availability

All the data in the article are available from the corresponding author upon reasonable request.

## Sample Availability

Samples of all the compounds are available from the authors.
